# Persistent organic pollutants and mortality in the United States, NHANES 1999–2011

**DOI:** 10.1186/s12940-017-0313-6

**Published:** 2017-10-10

**Authors:** Kristiann Fry, Melinda C. Power

**Affiliations:** Department of Epidemiology and Biostatistics, The George Washington University, 950 New Hampshire Avenue NW, 5th Floor, Washington DC, 20052 USA

**Keywords:** Persistent organic pollutants, Mortality, Flame retardants, Organochlorine pesticides, Polybrominated diphenyl ethers, PBDEs, Per- and polyfluoroalkyl substances, PFASs, Polychlorinated biphenyls, PCBs

## Abstract

**Background:**

Persistent organic pollutants (POPs) are environmentally and biologically persistent chemicals that include polybrominated diphenyl ethers (PBDEs), per- and polyfluoroalkyl substances (PFASs), polychlorinated biphenyls (PCBs), and organochlorine (OC) pesticides. Currently, data on the associations between exposure to POPs and the risk of mortality in the U.S. population is limited.

Our objective was to determine if higher exposure to POPs is associated with greater risk of all-cause, cancer, heart/cerebrovascular disease, or other-cause mortality.

**Methods:**

Analyses included participants aged 60 years and older from the 1999–2006 National Health and Nutrition Examination Surveys (NHANES). We included 483 participants for analyses of PBDEs, 1043 for PFASs, and 461 for PCBs, and 1428 for OC pesticides. Exposures to POPs were estimated using biomarkers measured in serum. Mortality status through December 31, 2011 was obtained from public-use, linked mortality files. We used Cox proportional hazard models to quantify the associations of interest. Where we observed an association, we explored effect modification by sex, body mass index, smoking status, and albuminuria. We also explored the combined effect of PBDEs and OC pesticides in the subsample of participants with measures of both analytes.

**Results:**

Serum measurements of PBDEs, PFASs, and PCBs were not clearly associated with increased all-cause or cause-specific mortality in older Americans. Beta-hexachlorocyclohexane was associated with an increased risk of all-cause mortality [HR per 1 SD increase =1.18, 95% CI = 1.01, 1.38]. Oxychlordane [HR = 1.15 95% CI 1.06, 1.25], p,p’-DDE [HR = 1.12, 95% CI = 1.02, 1.23], trans-nonachlor [HR = 1.11, 95% CI = 1.04, 1.18], and beta-hexachlorocyclohexane [HR = 1.25, 95% CI = 1.03, 1.52] were associated with increased risk of other-cause mortality. Exploratory analyses suggested associations between OC pesticides and other-cause mortality were modified by sex. Exploratory analyses also suggested that the combination of high PBDE and high OC pesticide exposure had a stronger than expected adverse effect on all-cause mortality.

**Conclusion:**

Higher exposure to beta-hexachlorocyclohexane, an OC pesticide, is associated with increased all-cause mortality and higher exposure to four OC pesticides is associated with increased non-cancer, non-heart/cerebrovascular disease mortality in U.S. adults 60 years or older. These associations may be modified by sex or exposure to other POPs.

**Electronic supplementary material:**

The online version of this article (10.1186/s12940-017-0313-6) contains supplementary material, which is available to authorized users.

## Background

Persistent organic pollutants (POPs) are chemicals that include polybrominated diphenyl ethers (PBDEs), per- and polyfluoroalkyl substances (PFASs), polychlorinated biphenyls (PCBs), and organochlorine (OC) pesticides. POPs persist environmentally and biologically and often bioaccumulate [1–5]; thus low-level exposure is widespread despite cessation or restrictions on manufacture and use, and POPs continue to be detectable in a large portion of the U.S population [6, 7]. However, we know relatively little about the health effects of exposure to these compounds, including their impact on mortality. Considering the prevalence, persistence, and bioaccumulation of POPs, an association with mortality in the U.S. population would have significant public health implications.

PBDEs are mixtures of chemicals that have been used as flame-retardants in many consumer products such as upholstery, plastics, and cabinets [3, 8]. Exposure to PBDEs often occur through ingesting contaminated dust, food, and breastmilk; touching contaminated soil or commercial products; and inhaling contaminated air [9, 10]. Some studies have suggested that exposure to PBDEs is associated with changes to neurodevelopment [11, 12], however, our understanding of the human health effects due to exposure is limited. To date, we are unaware of any studies assessing the association between PBDEs and mortality in the general population.

PFASs consist of a group of manufactured chemicals that repel water and oil, making them resistant to stains [4, 13]. Because of this characteristic, PFASs have been used for consumer products such as cookware, sofas, carpets, clothing, food packaging, paints, and cleaning products [4, 13]. Thus, exposure to PFASs can occur through contact with products treated with PFASs; contact with contaminated water, soil, and outdoor air near the industrial sites that manufacture or use PFASs; and consumption of contaminated food [14–17]. As with PBDEs, questions about the health effects related to exposure of PFASs at the levels that are normally found in the environment remain. Although existing studies often report conflicting findings, PFASs have been reported to increase cholesterol [18, 19] and risk of cancer [20], affect the immune system [21, 22], and decrease fertility [23]. However, as with PBDEs, whether non-occupational exposure to PFASs increases morality risk is unknown.

PCBs include manufactured mixtures of chlorinated compounds that were used as lubricants and coolants in electrical equipment, as well as other industrial applications such as plasticizers and pigments [1, 24]. The production of PCBs in the U.S. ended in 1979 because of their persistence in the environment and concern about potential health effects [24] and many countries worldwide eliminated the production and use of PCBs in 2004, in accordance with the Stockholm Convention [25]. However, because PCBs persist in the environment for many years, exposure to PCBs persists through use of contaminated electronic equipment and consumption of contaminated soil, air, water, food, and breastmilk [1, 26–28]. Increasing evidence suggests that low-level exposure to PCBs may have adverse health consequences [29–32] and the Environmental Protection Agency (EPA) has classified PCBs as probable human carcinogens [24]; however, whether PCB exposure increase risk of mortality in the general population remains uncertain. Two prior studies have used NHANES data to analyze associations of PCBs with mortality in the general population, however, they were limited by relatively short follow-up times and the omission of considering education or other measures of socioeconomic status as potential confounders [33, 34].

Finally, OC pesticides are a class of pesticides that were used because of their effectiveness against a wide variety of insects. OC pesticides that were once in common use include dichlorodiphenyltrichloroethane (DDT), oxychlordane, dieldrin, hexachlorobenzene, hexachlorocyclohexanes, and trans-nonachlor [26]. Due to their persistence in the environment and evidence of health effects, the EPA has restricted or cancelled the use of many OC pesticides [26]. Additionally, the Stockholm Convention eliminated or restricted the use of many OC pesticides throughout the world in 2004 [25]. However, these compounds continue to persist in the environment and DDT is still in use in malaria-afflicted areas. Thus, exposure to OC pesticides can occur through consumption of contaminated food, water and breastmilk; presence during application or manufacture; and touching or ingesting contaminated soil [35, 36]. Hexachlorocyclohexanes, hexachlorobenzene, heptachlor, DDT, and dichlorodiphenyldichloroethylene (DDE)—a metabolite of DDT—are classified by the EPA as probable human carcinogenic agents [37–40]. One prior study using NHANES data to assess the association between OC pesticide exposures and mortality in the general population found no significant overall association [34]. However, this study was limited by a relatively short follow-up time and the omission of education or other measures of socioeconomic status as covariates [41].

Given the sparsity of data on the associations between POPs and mortality, our objective was to determine if higher exposure to POPs—including individual PFASs, OC pesticides, and individual or summary measures of non-dioxin-like PCBs, dioxin-like PCBs, and PBDEs—are associated with greater risk of mortality in persons aged 60 years and older. We hypothesized that higher exposure to POPs increases the risk of mortality in persons aged 60 years and older.

## Methods

### Study population

NHANES is conducted by the National Center for Health Statistics (NCHS) to evaluate the health and nutritional status of adults and children in the United States. NHANES represents all noninstitutionalized civilians living within the United States and utilizes a complex, four-stage sample design [42]. Annually, NHANES collects data from approximately 5000 participants; data collected includes household interviews, physical examinations, and biological specimens for laboratory tests [42]. The laboratory tests are conducted on smaller, nationally representative subsamples of individuals from the total examined study population to reduce respondent burden and aid in the completion of exams [43]. The subsamples that are used for laboratory tests maintain the representative design of the survey because the subsamples are chosen at random [43]. Additional details are publicly available at: www.cdc.gov/nchs/nhanes. Our study used NHANES data for the survey periods of 1999–2006 to determine exposure levels and status of covariates. Different POPs were measured in different subsamples of survey cycles. Therefore, our study used data from 4 different subsamples with POPs measurements: OC pesticides from the 1999–2004 cycles, PBDEs and PCBs from the 2003–2004 cycle, and PFASs from the 2003–2006 cycles.

### Measurement of POP concentrations

The NHANES laboratory measured PBDE analytes in serum by performing solid phase extraction and isotope dilution gas chromatography high-resolution mass spectrometry (GC/IDHRMS), while PCB and OC pesticide analytes in serum were measured by high-resolution gas chromatography/isotope-dilution high-resolution mass spectrometry (HRGC/ID-HRMS); PFAS analytes were measured by using solid phase extraction-high performance liquid chromatography-turboionspray ionization-tandem mass spectrometry (SPE-HPLC-TCI-MS/MS). Details of the laboratory methods are publicly available at: https://wwwn.cdc.gov/nchs/nhanes/. We used the value of limit of detection (LOD)/√2 for all analyte measures that resulted in report of concentrations below the LOD. Additionally, we used the lipid-adjusted concentrations for analyses of PBDEs, OC pesticides, and PCBs in our primary analyses. To address the possibility of potentially influential outliers in our relatively small samples, we natural log transformed each exposure to normalize the distribution. We then applied the Extreme Studentized Deviate Multiple Outlier Procedure to identify and exclude outliers a priori [44]. This process resulted in the identification and exclusion of 11 observations from perfluorooctanoic acid (PFOA) measures, 7 from perfluorooctane sulfonic acid (PFOS) measures, 2 from trans-nonachlor measures, 5 from p,p’-DDE measures, 4 from oxychlordane measures, and 3 from β-hexachlorocyclohexane measures. After outlier removal, we standardized all analyte concentrations by subtracting the sample mean and dividing by the standard deviation.

### Mortality outcomes

We determined mortality status using the NCHS 2011 Public-use Linked Mortality Files. The length of follow-up time for each participant was determined using the NHANES study examination date and date of death, if dead, or end of follow-up, December 31, 2011. The cause of death coding follows the 10th revision of the International Statistical Classification of Diseases (ICD-10) [45]. For analyses, we categorized mortality as either all-cause, cancer (ICD codes: C00-C97), heart or cerebrovascular diseases (CBVD) (ICD codes: I00-I09, I11, I13, I20-I51, I60-I69), or other-cause (non-cancer, non-heart/cerebrovascular disease) [45].

### Statistical analyses

We included participants in our analysis if they were 60 years of age or older at the time of the NHANES interview. We excluded participants from analysis if all exposure analyte measurements were missing or if they were missing data on any of the following covariates included in our primary analyses: age, gender, race/ethnicity, education, or smoking status. Given the larger number of participants missing values for body mass index (BMI), family poverty income ratio (PIR), or alcohol consumption, we filled in missing values using mode single imputation for these three covariates, which are used in sensitivity analyses.

For PBDEs, we excluded a total of 52 participants (9.6%) for missing all PBDE analyte measures. We excluded an additional 4 (0.07%) participants for missing covariate data; thus, we included a total of 483 PBDE observations for analysis. Out of a total of 11 PBDE analytes, we analyzed the association between PBDE concentrations and mortality only for the 4 analytes that were above the LOD in greater than 90% of the sample: 2,2′,4,4′,5,5′-hexabromodiphenyl ether (PBDE-153); 2,2′,4,4′,6-pentabromodiphenyl ether (PBDE-100); 2,2′,4,4′-tetrabromodiphenyl ether (PBDE-47); and 2,2′,4,4′,5,5′-hexabromobiphenyl (PBB-153). Additionally, we created summary measures of all PBDE concentrations (ng/g), as well as a sum for the 4 PDBE analytes (ng/g) detectable in >90% of the sample by summing the concentrations for the observations that had all PDBE analyte data available (i.e., participants with measurements below LOD were included, however those with no measurement data reported were excluded).

For PFASs, we excluded a total of 71 participants (6.4%) for missing all PFAS analyte measures; we removed an additional 4 participants (0.4%) from analysis due to missing covariate data. After exclusions, there was a total of 1043 observations for PFAS analyte analysis. Out of the 12 PFAS analytes, we analyzed associations between PFAS concentrations and mortality for those 4 individual PFASs detected in greater than 90% of the population: PFOA, PFOS, perfluorohexane sulfonic acid (PFHxS), and perfluorononanoic acid (PFNA).

We excluded 94 participants (16.8%) for missing all PCB analyte measures; we excluded an additional 3 observations (0.5%) due to covariate data missing. The resulting population used for analysis included 461 observations. Instead of analyzing the individual PCB analytes (29 dioxin-like and 26 non-dioxin-like), we considered six summary measures: (1) exposure to all dioxin-like PCBs, (2) exposure to dioxin-like PCBs with concentrations above the LOD in more than 90% of the population, (3) exposure to all non-dioxin-like PCBs, (4) exposure to non-dioxin-like PCB concentrations above the LOD in more than 90% of the population, (5) toxicity of exposure to dioxin-like PCBs based on toxic equivalency values (TEQs), and (6) toxicity of exposure to dioxin-like PCBs measured above the LOD in greater than 90% of the population based on TEQs. Similar to the PBDE summary measures, participants with measurements below LOD were included; however, those with no measurement data on any component PCB were excluded. For dioxin-like PCBs, TEQs were calculated using the 2005 WHO toxic equivalency factors (TEFs) [46]. The serum concentrations of the dioxin-like PCBs were multiplied by their respective TEF and then summed.

For OC pesticides, we excluded a total of 107 participants (6.9%) because all analyte data was missing. We removed an additional 6 participants (0.4%) due to missing covariate resulting in 1428 eligible participants. Out of a total of 13 OC pesticide analytes, only those 4 with measurements above the LOD in greater than 90% of the population were considered for analyses for associations with mortality: Trans-nonachlor; p,p’-DDE; Oxychlordane; and β-hexachlorocyclohexane.

We assessed the associations between the analyte exposures described above and mortality by using Cox proportional hazard models. We considered the complex survey design in analyses by using the appropriate subsample weights, stratum, and primary sampling units per NHANES recommendation. For our primary analyses, we adjusted for age, gender, race/ethnicity, education and smoking. In sensitivity analyses we also considered two additional levels of adjustment: age-adjusted and adjusted for all the covariates in our primary model plus BMI, PIR, and alcohol use. Additionally, we conducted sensitivity analyses: (1) omitting survey weighting, (2) adjusting for lipids rather than using lipid-adjusted analyte value, and (3) including previously excluded potential outliers.

We also conducted exploratory analyses considering effect modification by sex (male/female), BMI (<25/≥25 kg/m^2^), smoking status (yes/no), and urine albumin creatinine ratio (ACR) (<30/≥30 mg/g) using a multiplicative interaction term where our primary analyses suggested presence of an association. In addition, given PBDEs and OC pesticides were analyzed in the same 1/3rd subsample in the 2003–2004 NHANES cycle, we also conducted exploratory analyses in the ~450 individuals with both measures to determine whether PBDEs and OC pesticide exposures had synergistic impact on mortality. Specifically, we dichotomized exposures for both PBDEs and each OC pesticide at the median and included both terms and their multiplicative interaction in a single model. (We were not able to conduct this analysis with combinations including PFASs or PCBS given their concentrations were measured in different subsamples.) All analyses were performed using SAS version 9.4.

### Human subjects protection issues

NHANES data were previously collected with the approval of the NCHS Research Ethics Review Board and anonymized before release to the public. All participants provided informed consent [42].

## Results

Among the 483 participants included for PBDE analyses, a total of 25.7% died during the follow-up; 7.1% were determined to be due to heart/cerebrovascular diseases, 6.7% due to cancer, and 11.9% due to other-causes (Table 1). In the PBDE sample, the median age at time of examination was 70.4 years and the majority of subjects were non-Hispanic white (82.2%). Slightly less than half were male (43.6%) and had some college education or above (47.1%), while slightly more than half smoked (55.5%), consumed alcohol (58.6%), or had a family PIR greater than 2.00 (61.7%). The mean measure for BMI at time of examination was 28.0 kg/m^2^. The median follow-up time was 6.4 years (minimum: 0.08 years, maximum: 8.9 years). The characteristics of the 1043 subjects used in analyses for PFASs, 461 for PCBs, and 1428 for OC pesticides were similar, with the exception of follow-up time and cumulative mortality, which varied based on which NHANES cycles had POPs measurements (Table 1). Unweighted characteristics of the study sample are available in Additional file 1: Table S1.

**Table 1:** Characteristics and outcome of adults participating in the 1999–2006 NHANES by POPs analyte sample

	PBDEs (*N* = 483) NHANES 2003–2004		PFASs (*N* = 1043) NHANES 2003–2006		PCBs (*N* = 461) NHANES 2003–2004		OC pesticides (*N* = 1428) NHANES 1999–2004	
	Estimate^a^	Std. Error of Estimate^a^	Estimate^a^	Std. Error of Estimate^a^	Estimate^a^	Std. Error of Estimate^a^	Estimate^a^	Std. Error of Estimate^a^
Age (years), median^b^	70.4	0.3	71.0	0.3	70.2	0.4	70.5	0.2
Body mass index (kg/m^2^), mean	28.0	0.2	28.2	0.2	28.2	0.3	28.0	0.2
Follow-Up Time (years), median	6.4	0.2	5.5	0.1	7.4	0.1	8.7	0.2
Gender, % Male	43.6%	2.1	44.5%	2.0	42.8%	2.5	43.5%	1.2
Ethnicity, %								
White, Non-Hispanic	82.2%	2.2	82.6%	2.1	82.8%	4	81.9%	1.6
Hispanic	5.3%	2.3	5.1%	1.1	5%	1.9	6.8%	1.5
Black, Non-Hispanic	7.6%	1.3	8%	1.1	8.5%	2.6	8.2%	1.1
Other	4.9%	1.2	4.3%	0.9	3.7%	1.2	3.2%	0.6
Family Poverty Income Ratio, %								
Less than/equal 2	38.3%	2.7	42.7%	2.8	43.7%	4.3	46.3%	1.9
Greater than 2	61.7%	2.7	57.3%	2.8	56.3%	4.3	53.7%	1.9
Education Level, %								
High school or less	25.8%	3.8	27.3%	2.3	30.9%	4.0	30.1%	1.7
High school graduate/GED	27.2%	2.4	31.0%	2.1	26.3%	2.4	27.7%	1.6
Some college or above	47.1%	4.9	41.7%	1.7	42.8%	3.9	42.2%	1.9
Smoker, % Yes	55.5%	2.2	54.5%	1.7	57.5%	3.9	55.3%	1.5
Consume Alcohol, % Yes	58.6%	4.0	65.9%	1.9	56.9%	6.2	61.7%	2.2
Mortality Status During Follow-Up, % Deceased								
All-cause	25.7%	1.8	22.2%	1.5	23.0%	2.6	34.2%	1.4
Heart/cerebrovascular diseases	7.1%	1.1	5.7%	0.6	7.0%	1.0	7.9%	0.7
Cancer	6.7%	1.0	4.5%	0.7	4.8%	1.5	7.9%	0.8
Other-cause	11.9%	1.9	12.0%	1.5	11.2%	1.9	18.2%	1.4

Notes: OC, organochlorine; PBDEs, polybrominated diphenyl ethers; PCBs, polychlorinated biphenyls; PFASs, per- and polyfluoroalkyl substances; POPs, persistent organic pollutants

^a^Weighted to consider complex survey design

^b^ Adults 60 years or older included in study; individuals aged 85 and over were re-coded by NHANES to 85 years of age

Table 2 shows the mean serum measurements for the analyte measures used for analyses and Additional file 1: Tables S2 to S5 summarize correlations between analytes of a given POP class. Four PBDE analytes were detected above the LOD in greater than 90% of the cohort population: PBDE-153, PBDE-100, PBDE-47, and PBB-153. The two PBDE summary measures (total PBDEs and PBDEs above LOD in >90% of the sample) were highly correlated (*r* = 0.99, *p* < 0.0001). Four PFAS analytes were above the LOD in greater than 90% of the participants: PFOA, PFOS, PFHxS, and PFNA. PFAS analytes were not strongly correlated; correlation coefficients ranged from 0.22 (*p* < 0.0001) to 0.63 (p < 0.0001). For PCBs, correlations between each of the summary measures considering all PCBs of a given type and the corresponding summaries restricted to those detected in >90% of the sample were high (*r* = 0.97 or higher, all *p* < 0.0001). The correlations between all dioxin-like PCBs and non-dioxin-like PCBs were lower than PBDE summaries with *r* = 0.80 (p < 0.0001). A total of 4 OC pesticide analytes were above the LOD in greater than 90% of the study group: β-hexachlorocyclohexane; p,p’-DDE; Oxychlordane; and Trans-nonachlor. Notably, of the OC pesticides, p,p’-DDE, had a much higher mean serum concentration compared to any other analyte. The correlations between OC pesticides ranged from 0.07 (*p* = 0.007) to 0.86 (p < 0.0001).

**Table 2:** Serum concentrations of analyzed POPs in adults 60 years or older

Exposure Measure	N	Median^a^	Std. Error of Median ^a^
PBDEs (ng/g of lipid)			
Σ PBDEs	436	53.5	4.1
Σ PBDEs above LOD in >90%	473	42.5	3.7
2,2′,4,4′,5,5′-hexabromodiphenyl ether (PBDE-153)	483	5.1	0.4
2,2′,4,4′,6-pentabromodiphenyl ether (PBDE-100)	483	3.6	0.3
2,2′,4,4′-tetrabromodiphenyl ether (PBDE-47)	474	19.7	2.0
2,2′,4,4′,5,5′-hexabromobiphenyl (PBB-153)	481	4.4	0.7
PFASs (ng/g)			
Perfluorooctanoic acid (PFOA)	1036	23.7	0.7
Perfluorooctane sulfonic acid (PFOS)	1032	4.3	0.2
Perfluorohexane sulfonic acid (PFHxS)	1043	2.0	0.1
Perfluorononanoic acid (PFNA)	1043	1.0	0.07
PCBs (ng/g of lipid)			
Σ Dioxin-like PCBs	380	57.5	2.5
Σ Dioxin-like PCBs above LOD in >90%	414	58.0	2.4
Σ Non-dioxin-like PCBs	405	284.3	10.1
Σ Non-dioxin-like PCBs above LOD in >90%	413	281.7	10.4
Σ TEQ Dioxin-like PCBs	385	0.03	0.001
Σ TEQ Dioxin-like PCBs above LOD in >90%	420	0.01	0.001
OC Pesticides (ng/g of lipid)			
β-hexachlorocyclohexane	1397	25.1	1.1
p,p’-DDE	1411	666.1	33.0
Oxychlordane	1312	29.0	0.9
Trans-nonachlor	1404	44.8	1.7

Notes: DDE, dichlorodiphenyldichloroethylene; LOD, limit of detection; OC, organochlorine; PBDEs, polybrominated diphenyl ethers; PCBs, polychlorinated biphenyls; PFASs, per- and polyfluoroalkyl substances; POPs, persistent organic pollutants; TEQ, toxic equivalency values

^a^ weighted to consider complex survey design

**Fig. 1:**
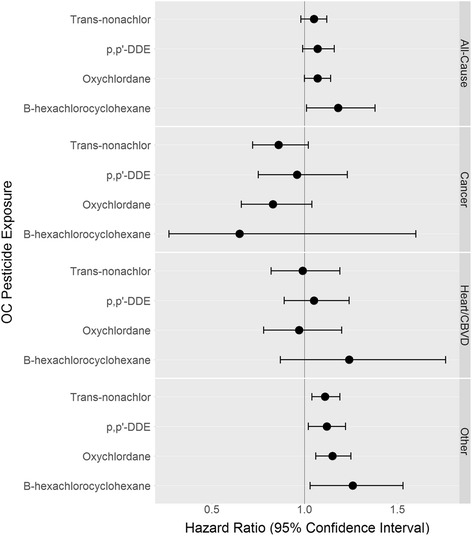
Associations^a^ between a one standard deviation unit increase in serum OC pesticide measures and mortality. Note: CBVD, cerebrovascular disease; DDE, dichlorodiphenyldichloroethylene; Heart, heart disease; OC, organochlorine. ^a^ Adjusted for age, gender, race/ethnicity, education, and smoking status

Table 3 summarizes the association between a one standard deviation unit increase in serum measurements and all-cause mortality, adjusting for age, gender, race/ethnicity, education, and smoking status. Higher exposure to PBDE-153 was associated with increased risk of all-cause mortality (HR: 1.07 per 1 SD unit increase in lipid-adjusted serum concentrations, 95% CI: 1.00, 1.14). There was little evidence to support associations between PFASs, PCBs, and the other PBDE measures with all-cause mortality. While the OC pesticides appeared to be associated with increased risk of all-cause mortality, only the association between β-hexachlorocyclohexane and all-cause mortality achieved statistical significance (HR:1.18 per 1 SD unit increase in lipid-adjusted serum concentrations 95%CI:1.01, 1.38). Exposure to PBDEs, PFASs, and PCBs were not associated with cause-specific mortality (Additional file 1: Tables S6-S8); however, higher exposure to all 4 OC pesticides was associated with increased risk of other-cause, but not cardiovascular or cancer mortality (Fig. 1, Additional file 1: Table S9). The results were materially unchanged with additional adjustment (Additional file 1: Tables S6-S9) and across all sensitivity analyses (Additional file 1: Tables S10-S13), with the exception that we no longer see an association between β-hexachlorocyclohexane and either all-cause or other-cause mortality in sensitivity analyses when we do not exclude potentially influential outliers.

**Table 3:** Associations between a one standard deviation unit increase in serum POP measures and all-cause mortality

Exposure	N	Hazard Ratio (95% CI)^a^	p-value^a^
PBDEs (ng/g of lipid)			
Σ PBDEs	436	1.07 (0.90, 1.27)	0.42
Σ PBDEs above LOD in >90%	473	1.10 (0.93, 1.30)	0.24
2,2′,4,4′,5,5′-hexabromodiphenyl ether (PBDE-153)	483	1.07 (1.00, 1.14)	0.06
2,2′,4,4′,6-pentabromodiphenyl ether (PBDE-100)	483	1.05 (0.91, 1.23)	0.47
2,2′,4,4′-tetrabromodiphenyl ether (PBDE-47)	474	1.10 (0.91, 1.33)	0.29
2,2′,4,4′,5,5′-hexabromobiphenyl (PBB-153)	482	1.04 (0.89, 1.21)	0.63
PFASs (ng/g)			
Perfluorooctane sulfonic acid (PFOS)	1036	0.91 (0.80, 1.03)	0.12
Perfluorooctanoic acid (PFOA)	1032	0.93 (0.82, 1.06)	0.27
Perfluorononanoic acid (PFNA)	1043	0.92 (0.80, 1.07)	0.26
Perfluorohexane sulfonic acid (PFHxS)	1043	0.88 (0.72, 1.08)	0.21
PCBs (ng/g of lipid)			
Σ Dioxin-like PCBs	380	0.94 (0.74, 1.18)	0.55
Σ Dioxin-like PCBs above LOD in >90%	414	0.91 (0.77, 1.08)	0.27
Σ Non-dioxin-like PCBs	405	0.94 (0.72, 1.22)	0.61
Σ Non-dioxin-like PCBs above LOD in >90%	413	0.97 (0.76, 1.24)	0.81
Σ TEQ Dioxin-like PCBs	385	1.01 (0.78, 1.31)	0.92
Σ TEQ Dioxin-like PCBs above LOD in >90%	420	0.99 (0.80, 1.23)	0.92
OC Pesticides (ng/g of lipid)			
Trans-nonachlor	1404	1.05 (0.98, 1.12)	0.17
p,p’-DDE	1411	1.07 (0.99, 1.16)	0.09
Oxychlordane	1312	1.07 (1.00, 1.14)	0.07
β-hexachlorocyclohexane	1397	1.18 (1.01, 1.38)	0.04

Notes: CI, confidence interval; DDE, dichlorodiphenyldichloroethylene; LOD, limit of detection; OC, organochlorine; PBDEs, polybrominated diphenyl ethers; PCBs, polychlorinated biphenyls; PFASs, per- and polyfluoroalkyl substances; POPs, persistent organic pollutants; TEQ, toxic equivalency values

^a^ Adjusted for age, gender, race/ethnicity, education, and smoking status; materially unchanged with additional adjustment for alcohol consumption, BMI, and poverty income ratio

As we observed statistically significant main effects for β-hexachlorocyclohexane with all-cause mortality and all four OC pesticide analytes with other-cause mortality, we proceeded to explore whether sex, BMI, smoking status, or ACR modified these associations; results are summarized in Table 4. Associations were uniformly stronger among overweight individuals, although there was little statistical support for effect modification with the exception of the association between oxychlordane and other-cause mortality (*p* = 0.04). There was little evidence of effect modification by smoking status or ACR. Finally, the association between OC exposure and other-cause mortality was stronger in men for trans-nonachlor, p,p’-DDE, and oxychlordane, but was stronger in women for β-hexachlorocyclohexane. However, there was no support for effect modification by sex when considering the association between β-hexachlorocyclohexane and all-cause mortality.

**Table 4:** Summary of exploratory analyses considering effect modification by BMI, smoking status, and sex where main effects were present

Mortality/Exposure	N	Hazard Ratio (95% CI)^a^		p-value for interaction	Hazard Ratio (95% CI)^a^		p-value for interaction
		BMI < 25	BMI ≥ 25		Non-Smoker	Current Smoker	
All-Cause							
β-hexachlorocyclohexane	1397	1.06 (0.82, 1.37)	1.36 (1.15, 1.62)	0.08	1.15 (0.98, 1.34)	1.36 (1.02, 1.8)	0.24
Other-cause							
Trans-nonachlor	1404	1.07 (0.98, 1.17)	1.15 (1.06, 1.26)	0.18	1.1 (1.04, 1.16)	1.14 (0.98, 1.33)	0.59
p,p’-DDE	1411	1.05 (0.74, 1.47)	1.13 (1.05, 1.22)	0.64	1.15 (1.05, 1.25)	1.03 (0.82, 1.3)	0.39
Oxychlordane	1312	1.09 (0.99, 1.2)	1.25 (1.12, 1.4)	0.04	1.12 (1.04, 1.2)	1.27 (1.07, 1.51)	0.12
β-hexachlorocyclohexane	1397	1.21 (0.96, 1.53)	1.35 (1.12, 1.63)	0.38	1.25 (1.02, 1.54)	1.3 (0.85, 1.98)	0.87
Mortality/Exposure	N	Hazard Ratio (95% CI)^a^		p-value for interaction	Hazard Ratio (95% CI)^a^		p-value for interaction
		Female	Male		ACR < 30	ACR ≥ 30	
All-Cause							
β-hexachlorocyclohexane	1397	1.22 (0.97, 1.54)	1.16 (0.96, 1.4)	0.71	1.16 (0.98, 1.39)	1.35 (1.08, 1.68)	0.30
Other-cause							
Trans-nonachlor	1404	1.07 (0.99, 1.15)	1.19 (1.07, 1.33)	0.07	1.08 (1, 1.17)	1.18 (1.01, 1.37)	0.28
p,p’-DDE	1411	1.05 (0.9, 1.23)	1.38 (1.13, 1.69)	0.01	1.1 (0.97, 1.24)	1.17 (0.98, 1.39)	0.52
Oxychlordane	1312	1.11 (1.02, 1.19)	1.37 (1.1, 1.7)	0.05	1.13 (1.04, 1.22)	1.18 (0.98, 1.41)	0.64
β-hexachlorocyclohexane	1397	1.46 (1.15, 1.85)	1.11 (0.89, 1.39)	0.05	1.26 (1.01, 1.56)	1.37 (0.96, 1.96)	0.67

^a^Weighted to consider complex survey design, adjusted for age, gender, race/ethnicity, education, and smoking status

In exploratory analyses, there was little evidence of interaction between PBDEs and OC pesticide exposure on cancer or cardiovascular/cerebrovascular mortality. However, we did observe a marginally significant interaction between PBDEs with p,p’-DDE and oxychlordane whereby those with high exposure to both PBDEs and either OC pesticide had higher than expected other-cause mortality (Table 5). While similar trends were observed for trans-nonachlor or β-hexachlorocyclohexane there was little statistical support for effect modification in these analyses. While we observed statistically significant *p*-values for the interaction of PBDEs with p,p’-DDE, the pattern of association observed for all-cause mortality was not repeated. There was no statistical support for interaction with the other OC pesticides on other-cause mortality. Of note, our category-specific estimates are often imprecise given small numbers.

**Table 5:** Summary of exploratory analyses considering synergism of exposure to PBDEs and OC pesticides on all-cause and other-cause mortality

		HR (95%CI)^a^, all-cause mortality Σ PBDEs above LOD in >90%			HR (95%CI)^a^, other-cause mortality Σ PBDEs above LOD in >90%		
		Less than median	Higher than median	interaction *p*-value	Less than median	Higher than median	interaction *p*-value
Trans-nonachlor	Less than median	1.0 (Ref.)	1.08 (0.58, 2.00)	0.09	1.0 (Ref.)	0.47 (0.12, 1.86)	0.24
	Higher than median	1.07 (0.67, 1.70)	1.96 (1.11, 3.43)		0.95 (0.47, 1.94)	1.00 (0.52, 1.93)	
p,p’-DDE	Less than median	1.0 (Ref.)	1.10 (0.63, 1.94)	0.08	1.0 (Ref.)	0.37 (0.17, 0.81)	0.01
	Higher than median	0.84 (0.46, 1.54)	1.63 (1.03, 2.59)		0.93 (0.42, 2.08)	1.06 (0.46, 2.42)	
Oxychlordane	Less than median	1.0 (Ref.)	0.90 (0.40, 2.04)	0.14	1.0 (Ref.)	0.88 (0.39, 1.97)	0.29
	Higher than median	0.87 (0.50, 1.51)	1.60 (1.00, 2.54)		0.34 (0.05, 2.49)	0.96 (0.54, 1.70)	
β-hexachlorocyclohexane	Less than median	1.0 (Ref.)	1.13 (0.61, 2.10)	0.26	1.0 (Ref.)	0.60 (0.17, 2.1)	0.49
	Higher than median	0.78 (0.41, 1.48)	1.43 (0.86, 2.74)		0.78 (0.36, 1.70)	0.76 (0.37, 1.54)	

^a^Adjusted for age, gender, race/ethnicity, education, and smoking status

## Discussion

Our study found that serum measurements of PBDEs, PFASs, and PCBs are not clearly associated with increased mortality in the U.S. population aged 60 years or older. β-hexachlorocyclohexane, an OC pesticide, was associated with an increased risk of all-cause mortality. All four OC pesticides detectable in >90% of the sample (oxychlordane; p,p’-DDE; Trans-nonachlor; and β-hexachlorocyclohexane) were associated with increased risk of other-cause (non-cancer, non-cardiovascular) mortality. Exploratory analyses suggest these associations may be modified by sex, BMI, or PBDE exposures, but this requires further confirmation in an independent sample.

To our knowledge, this study was the first to examine the effects of PBDE exposures on mortality in a human sample. Prior studies of the associations between exposure to PFASs and mortality were conducted in occupational settings and report either no association or lower mortality in workers exposed to PFASs [47–50]. Our results were similar; while our point estimates suggested PFASs were associated with decreased mortality these results were not statistically significant. Multiple prior studies report elevated risk of cancer-related mortality with PCB exposure in occupational and poisoning settings, although these results are not conclusive given concerns about unclear dose-response relationships and residual confounding [51–58]. Prior studies in NHANES using summaries of PCB exposures from 1999 to 2004 report associations between total PCB exposures and excess cardiovascular mortality, but not all-cause or cancer-related mortality, among adults 70 years or older over a mean of 4.1 years of follow-up [41] and associations between higher exposure to dioxin-like compounds and all-cause mortality in adults ages 40 or older over a mean of 4.6 years of follow-up [33]. Our results conflict with these prior findings as we found no association between PCBs and all-cause or cause-specific analyses and may be related to lack of adjustment for education or other measures of socioeconomic status, shorter follow-up time, and consideration of a smaller, different set of compounds in prior studies. Finally, prior studies of occupational OC pesticide exposure and mortality generally report found lower than expected rates of all-cause mortality in exposed persons, but likely suffer from healthy-worker bias [59–61] A prior study using data from NHANES reported no association between exposure to OC pesticides and all-cause mortality, cancer mortality, or cardiovascular mortality over a mean 4.1 year follow-up period in persons 70 years and older [41], but did not consider other-cause mortality. Thus, our finding of increased risk of other-cause (non-cancer, non-cardiovascular) mortality with greater exposure OC pesticides is novel. Given the heterogeneous nature of “other-cause” mortality and the wide-ranging causes of death that are ultimately attributable to underlying diabetes or dementia, it is plausible that our results for OC pesticide exposure and other-cause mortality is due to associated increased risk of diabetes or dementia. Several recent studies have linked OC pesticide exposure to incident diabetes or markers indicative of increased risk of diabetes [62–64] as well as to elevated risk for cognitive decline or dementia [65–67].

While our report of exploratory analyses considering the combined impact of PBDEs and OC pesticides is novel, several prior studies suggest modification of the POPs-mortality association by BMI, smoking, or ACR. While not statistically significantly in our analyses, the suggestion of greater mortality among overweight and obese individuals with high OC pesticide exposure is opposite than reported previously. [41, 68] Conversely, while we did not observe significant effect modification by ACR, our point estimates were often consistent with the prior report of greater mortality among those with high ACR and high OC pesticide exposure [69] or among current smokers with high OC pesticide exposure [70]. The suggestion that sex modifies the OC pesticide other-cause mortality association is potentially plausible given potential endocrine-disruption related effects of exposure [71], but requires further confirmation in other samples.

There are limitations to our study. First, our sample size for each analysis was relatively small, due to data availability or concerns about combining multiple years of exposure data in the face of changing lists of analytes assessed in different cycles and potential time trends in exposure. This limited our ability to detect small effect and our ability to further investigate the association with more refined cause-specific mortality categories. Similarly, although motivated by evidence of effect modification in other settings [34, 68–70], we consider our analyses of effect modification to be exploratory given our small sample. Future work in larger samples will be required to examine the effect of exposure within potentially susceptible sub-populations. We also view our analyses of the combined effect of PBDEs and OC pesticides to be exploratory, and were unable to consider combined effects of multiple classes of POPs, as PCBs, PFASs, and PBDEs/OC pesticidess were measured in different subsamples. As with any epidemiologic study, we cannot definitively rule out the possibility of bias or chance. However, we adjusted our analyses for many possible confounders, minimizing the potential for residual confounding. We used a nationally-representative sample and evaluated associations with mortality, limiting the potential for selection bias. Finally, as our exposure and outcome are objective outcomes, and mortality was assessed independently of exposure status, we expect minimal impact of measurement error and that any resulting bias would be towards the null.

Our study also has strengths. The data is from NHANES and is a nationally representative study [42], thus our findings are generalizable to the older U.S. population as a whole. Several aspects of our primary analyses were novel, including examination of the association of PBDEs with mortality and consideration of associations between POPs and non-CVD, non-cancer mortality. We were able to address several of the potential limitations of the previous literature, including addressing potential confounding by education and socioeconomic status. Additionally, our study benefited from relatively long follow-up times, ranging from 9 to 13 years, which minimizes the potential impact of reverse causation, whereby impending death impacts POPs exposure levels.

## Conclusions

Our study provides evidence of an adverse association between exposure to OC pesticides and non-cancer, non-heart/cerebrovascular disease mortality in adults 60 years or older in the U.S. This association may be modified by several factors, including exposure to other POPs. Additional research to confirm this finding and determine how OC pesticides may impact other-cause mortality is warranted.

## Additional file

Additional file 1: Table S1 Unweighted characteristics of the study sample. **Tables S2-S5.** Correlation coefficients between within-class POP analytes. **Tables S6-S9.** Associations between a one standard deviation unit increase in serum POP measures and all-cause, cancer, heart/cerebrovascular diseases, and other-cause mortality with varying levels of adjustment. **Tables S10-S13.** Sensitivity analyses for associations between a one standard deviation unit increase in serum POP measures and all-cause, cancer, heart/cerebrovascular diseases, and other-cause mortality. (DOCX 79 kb)

## Abbreviations

ACR: Albumin creatinine ratio
BMI: Body mass index
CBVD: Cerebrovascular diseases
DDE: Dichlorodiphenyldichloroethylene
DDT: Dichlorodiphenyltrichloroethane
LOD: Limit of detection
NCHS: National Center for Health Statistics
NHANES: National Health and Nutrition Examination Surveys
OC: Organochlorine
PBB-153: 2,2′,4,4′,5,5′-hexabromobiphenyl
PBDE-100: 2,2′,4,4′,6-pentabromodiphenyl ether
PBDE-153: 2,2′,4,4′,5,5′-hexabromodiphenyl ether
PBDE-47: 2,2′,4,4′-tetrabromodiphenyl ether
PBDEs: Polybrominated diphenyl ethers
PCBs: Polychlorinated biphenyls
PFASs: Per- and polyfluoroalkyl substances
PFHxS: Perfluorohexane sulfonic acid
PFNA: Perfluorononanoic acid
PFOA: Perfluorooctanoic acid
PFOS: Perfluorooctane sulfonic acid
PIR: Family poverty income ratio
POPs: Persistent organic pollutants
TEFs: Toxic equivalency factors
TEQs: Toxic equivalency values
