# Supplementary material for: Persistent organic pollutants and mortality in the United States, NHANES 1999–2011
Source: Environ Health. 2017 Oct 10;16:105. doi: 10.1186/s12940-017-0313-6 (PMC5634885; doi:10.1186/s12940-017-0313-6)
Supplement: Additional file 1: Table S1 — Unweighted characteristics of the study sample. Tables S2-S5. Correlation coefficients between within-class POP analytes. Tables S6-S9. Associations between a one standard deviation unit increase in serum POP measures and all-cause, cancer, heart/cerebrovascular diseases, and other-cause mortality with varying levels of adjustment. Tables S10-S13. Sensitivity analyses for associations between a one standard deviation unit increase in serum POP measures and all-cause, cancer, heart/cerebrovascular diseases, and other-cause mortality. (DOCX 79 kb) [file 12940_2017_313_MOESM1_ESM.docx]

**Persistent Organic Pollutants and Mortality in the United States, NHANES 1999-2011**

Kristiann Fry^a^ and Melinda C. Power^a^

^a^Department of Epidemiology and Biostatistics, The George Washington University, Washington, DC, United States

**Supplemental Material:**

**Table S1:** Unweighted characteristics of the study sample

**Tables S2-S5:** Correlation coefficients between within-class POP analytes

**Tables S6-S9:**  Associations between a one standard deviation unit increase in serum POP measures and all-cause, cancer, heart/cerebrovascular diseases, and other-cause mortality with varying levels of adjustment

**Tables S10-S13:**  Sensitivity analyses for associations between a one standard deviation unit increase in serum POP measures and all-cause, cancer, heart/cerebrovascular diseases, and other-cause mortality

**Table S1. Unweighted characteristics and outcome of adults 60 years or older participating in the 1999-2006 NHANES by POP analyte group**

|  | **PBDEs**  (N=483) NHANES 2003-2004 | **PFASs**  (N=1043) NHANES 2003-2006 | **PCBs**  (N=461) NHANES 2003-2004 | **OC pesticides** (N=1428) NHANES 1999-2004 |
| --- | --- | --- | --- | --- |
|  | **Estimate (SD)** | **Estimate (SD)** | **Estimate (SD)** | **Estimate (SD)** |
| **Age (years), median^a^** | 72.0 (8.0) | 70.1 (8.1) | 71.0 (8.1) | 71.0 (7.9) |
| **Body mass index (kg/m^2^), mean** | 28.2 (5.4) | 28.2 (5.7) | 28.2 (5.1) | 28.2 (5.4) |
| **Follow-Up Time (years), median** | 7.5 (2.1) | 6.4 (2.1) | 7.5 (2.3) | 8.6 (3.0) |
| **Gender, % Male** | 48.5% | 52.1% | 50.1% | 47.7% |
| **Ethnicity, %** |  |  |  |  |
| White, Non-Hispanic | 60.5% | 59.9% | 63.6% | 58.8% |
| Hispanic | 22.6% | 20.0% | 20.2% | 24.2% |
| Black, Non-Hispanic | 13.5% | 17.1% | 12.8% | 14.6% |
| Other | 3.5% | 3.0% | 3.5% | 2.5% |
| **Family Poverty Income Ratio, %** | | | | |
| Less than/equal 2 | 50.1% | 52.5% | 54.5% | 53.6% |
| Greater than 2 | 49.9% | 47.5% | 45.6% | 46.4% |
| **Education Level, %** |  |  |  |  |
| High school or less | 38.9% | 39.1% | 40.6% | 42.9% |
| High school graduate/GED | 23.0% | 25.4% | 24.1% | 22.3% |
| Some college or above | 38.1% | 35.5% | 35.4% | 34.8% |
| **Smoker, % Yes** | 54.9% | 53.8% | 60.1% | 53.6% |
| **Consume Alcohol, % Yes** | 63.2% | 66.0% | 61.6% | 63.5% |
| **Mortality Status During Follow-Up, % Deceased** | | | | |
| All-cause | 31.1% | 26.1% | 30.4% | 37.3% |
| Heart/cerebrovascular diseases | 9.9% | 7.0% | 8.7% | 9.8% |
| Cancer | 7.7% | 5.3% | 6.5% | 8.3% |
| Other-cause | 13.5% | 13.8% | 15.2% | 18.8% |
| Notes: OC, organochlorine; PBDEs, polybrominated diphenyl ethers; PCBs, polychlorinated biphenyls; PFASs, per- and polyfluoroalkyl substances; POPs, persistent organic pollutants | | | | |
| ^a^ Individuals aged 85 and over were recoded by NHANES to 85 years of age. | | | | |

| **Exposure** | **Pearson Correlation (p-value)** | | | | | |
| --- | --- | --- | --- | --- | --- | --- |
|  | **Σ PBDEs** | **Σ PBDEs above LOD in >90%** | **PBDE-153** | **PBDE-100** | **PBDE-47** | **PBB-153** |
| **Σ PBDEs** | 1 | 0.99 (<.0001) | 0.73 (<.0001) | 0.93 (<.0001) | 0.96 (<.0001) | 0.16 (0.0009) |
| **Σ PBDEs above LOD in >90%** | 0.99 (<.0001) | 1 | 0.74 (<.0001) | 0.94 (<.0001) | 0.95 (<.0001) | 0.17 (0.0002) |
| **PBDE-153** | 0.73 (<.0001) | 0.74 (<.0001) | 1 | 0.74 (<.0001) | 0.52 (<.0001) | 0.05 (0.2487) |
| **PBDE-100** | 0.93 (<.0001) | 0.94 (<.0001) | 0.74 (<.0001) | 1 | 0.87 (<.0001) | 0.03 (0.4671) |
| **PBDE-47** | 0.96 (<.0001) | 0.95 (<.0001) | 0.52 (<.0001) | 0.87 (<.0001) | 1 | 0.03 (0.4726) |
| **PBB-153** | 0.16 (0.0009) | 0.17 (0.0002) | 0.05 (0.2487) | 0.03 (0.4671) | 0.03 (0.4726) | 1 |
| Notes: LOD, limit of detection; PBB-153, 2,2',4,4',5,5'-hexabromobiphenyl; PBDE, polybrominated diphenyl ether; PBDE-153, 2,2',4,4',5,5'-hexabromodiphenyl ether; PBDE-100, 2,2',4,4',6-pentabromodiphenyl ether; PBDE-47, 2,2',4,4'-tetrabromodiphenyl ether | | | | | | |

**Table S2. Pearson Correlation Coefficients for PBDE summary measures and analytes**

**Table S3. Pearson Correlation Coefficients for PFAS analytes**

| **Exposure** | **Pearson Correlation (p-value)** | | | |
| --- | --- | --- | --- | --- |
|  | **PFOS** | **PFOA** | **PFNA** | **PFHxS** |
| **PFOS** | 1 | 0.63 (<.0001) | 0.45 (<.0001) | 0.30 (<.0001) |
| **PFOA** | 0.63 (<.0001) | 1 | 0.51 (<.0001) | 0.33 (<.0001) |
| **PFNA** | 0.45 (<.0001) | 0.51 (<.0001) | 1 | 0.22 (<.0001) |
| **PFHxS** | 0.30 (<.0001) | 0.33 (<.0001) | 0.22 (<.0001) | 1 |
| Notes: PFAS, per- and polyfluoroalkyl substance; PFHxS, Perfluorohexane sulfonic acid; PFNA, Perfluorononanoic acid; PFOS, Perfluorooctane sulfonic acid; PFOA, Perfluorooctanoic acid | | | | |

**Table S4. Pearson Correlation Coefficients for PCB summary measures**

| **Exposure** | **Pearson Correlation (p-value)** | | | | | |
| --- | --- | --- | --- | --- | --- | --- |
|  | **Σ Dioxin-like PCBs** | **Σ Dioxin-like PCBs above LOD in >90%** | **Σ Non-dioxin-like PCBs** | **Σ Non-dioxin-like PCBs above LOD in >90%** | **Σ TEQ Dioxin-like PCBs** | **Σ TEQ Dioxin-like PCBs above LOD in >90%** |
| **Σ Dioxin-like PCBs** | 1 | 0.99 (<.0001) | 0.80 (<.0001) | 0.80 (<.0001) | 0.80 (<.0001) | 0.86 (<.0001) |
| **Σ Dioxin-like PCBs above LOD in >90%** | 0.99 (<.0001) | 1 | 0.79 (<.0001) | 0.78 (<.0001) | 0.80 (<.0001) | 0.80 (<.0001) |
| **Σ Non-dioxin-like PCBs** | 0.80 (<.0001) | 0.79 (<.0001) | 1 | 0.99 (<.0001) | 0.69 (<.0001) | 0.71 (<.0001) |
| **Σ Non-dioxin-like PCBs above LOD in >90%** | 0.80 (<.0001) | 0.78 (<.0001) | 0.99 (<.0001) | 1 | 0.69 (<.0001) | 0.71 (<.0001) |
| **Σ TEQ Dioxin-like PCBs** | 0.80 (<.0001) | 0.80 (<.0001) | 0.69 (<.0001) | 0.69 (<.0001) | 1 | 0.96 (<.0001) |
| **Σ TEQ Dioxin-like PCBs above LOD in >90%** | 0.86 (<.0001) | 0.80 (<.0001) | 0.71 (<.0001) | 0.71 (<.0001) | 0.96 (<.0001) | 1 |
| Notes: LOD, limit of detection; PCB, polychlorinated biphenyl; TEQ, toxic equivalency values | | | | | | |

**Table S5. Pearson Correlation Coefficients for OC pesticide analytes**

| **Exposure** | **Pearson Correlation (p-value)** | | | |
| --- | --- | --- | --- | --- |
|  | **Trans-nonachlor** | **p,p'-DDE** | **Oxychlordane** | **β-hexachlorocyclohexane** |
| **Trans-nonachlor** | 1 | 0.24 (<.0001) | 0.86 (<.0001) | 0.07 (0.0073) |
| **p,p'-DDE** | 0.24 (<.0001) | 1 | 0.24 (<.0001) | 0.22 (<.0001) |
| **Oxychlordane** | 0.86 (<.0001) | 0.24 (<.0001) | 1 | 0.09 (0.0018) |
| **β-hexachlorocyclohexane** | 0.07 (0.0073) | 0.22 (<.0001) | 0.09 (0.0018) | 1 |
| Notes: DDE, dichlorodiphenyldichloroethylene; OC, organochlorine | | | | |

**Table S6. Associations between a one standard deviation unit increase in serum PBDE measures and all-cause, cancer, heart/cerebrovascular diseases, and other-cause mortality with varying levels of adjustment**

| **Mortality/Exposure** (ng/g of lipid) | **N** | **Age Adjusted HR (95% CI)^a^** | **p-value^a^** | **Primary Analysis HR (95% CI)^ab^** | **p-value^ab^** | **Additionally Adjusted HR (95% CI)^ac^** | **p-value^ac^** |
| --- | --- | --- | --- | --- | --- | --- | --- |
| **All-Cause** | | | | | | | |
| Σ PBDEs above LOD in >90% | 473 | 1.12 (0.95, 1.33) | 0.17 | 1.10 (0.93, 1.30) | 0.24 | 1.10 (0.92, 1.31) | 0.27 |
| Σ PBDEs | 436 | 1.08 (0.91, 1.28) | 0.34 | 1.07 (0.90, 1.27) | 0.42 | 1.06 (0.89, 1.28) | 0.48 |
| 2,2',4,4',5,5'-hexabromodiphenyl ether (PBDE-153) | 483 | 1.08 (1.003, 1.17) | 0.04 | 1.07 (1.00, 1.14) | 0.06 | 1.06 (0.99, 1.13) | 0.08 |
| 2,2',4,4',6-pentabromodiphenyl ether (PBDE-100) | 483 | 1.07 (0.92, 1.25) | 0.36 | 1.05 (0.91, 1.23) | 0.47 | 1.05 (0.90, 1.24) | 0.49 |
| 2,2',4,4'-tetrabromodiphenyl ether (PBDE-47) | 474 | 1.13 (0.94, 1.35) | 0.19 | 1.10 (0.91, 1.33) | 0.29 | 1.10 (0.90, 1.34) | 0.32 |
| 2,2',4,4',5,5'-hexabromobiphenyl (PBB-153) | 482 | 1.03 (0.89, 1.20) | 0.69 | 1.04 (0.89, 1.21) | 0.63 | 1.03 (0.88, 1.20) | 0.73 |
| **Cancer** | | | | | | | |
| Σ PBDEs | 436 | 1.08 (0.82, 1.42) | 0.56 | 1.06 (0.82, 1.37) | 0.65 | 1.06 (0.81, 1.40) | 0.65 |
| Σ PBDEs above LOD in >90% | 473 | 1.18 (0.93, 1.50) | 0.16 | 1.15 (0.89, 1.49) | 0.26 | 1.16 (0.88, 1.52) | 0.28 |
| 2,2',4,4',5,5'-hexabromodiphenyl ether (PBDE-153) | 483 | 0.97 (0.79, 1.19) | 0.74 | 0.95 (0.80, 1.12) | 0.52 | 0.94 (0.79, 1.13) | 0.49 |
| 2,2',4,4',6-pentabromodiphenyl ether (PBDE-100) | 483 | 1.11 (0.88, 1.39) | 0.35 | 1.09 (0.87, 1.36) | 0.45 | 1.09 (0.86, 1.39) | 0.46 |
| 2,2',4,4'-tetrabromodiphenyl ether (PBDE-47) | 474 | 1.21 (0.96, 1.54) | 0.10 | 1.19 (0.91, 1.55) | 0.19 | 1.19 (0.89, 1.61) | 0.22 |
| 2,2',4,4',5,5'-hexabromobiphenyl (PBB-153) | 482 | 1.06 (0.89, 1.26) | 0.47 | 1.06 (0.87, 1.29) | 0.53 | 1.06 (0.87, 1.28) | 0.56 |
| **Heart/cerebrovascular diseases** | | | | | | | |
| Σ PBDEs | 436 | 1.11 (0.95, 1.30) | 0.17 | 1.09 (0.95, 1.25) | 0.20 | 1.10 (0.94, 1.28) | 0.21 |
| Σ PBDEs above LOD in >90% | 473 | 1.15 (0.98, 1.36) | 0.09 | 1.12 (0.97, 1.30) | 0.12 | 1.13 (0.96, 1.32) | 0.12 |
| 2,2',4,4',5,5'-hexabromodiphenyl ether (PBDE-153) | 483 | 1.14 (0.96, 1.34) | 0.12 | 1.11 (0.94, 1.31) | 0.19 | 1.11 (0.94, 1.31) | 0.21 |
| 2,2',4,4',6-pentabromodiphenyl ether (PBDE-100) | 483 | 1.11 (0.96, 1.28) | 0.15 | 1.08 (0.94, 1.24) | 0.25 | 1.09 (0.94, 1.25) | 0.23 |
| 2,2',4,4'-tetrabromodiphenyl ether (PBDE-47) | 474 | 1.12 (0.93, 1.35) | 0.23 | 1.10 (0.92, 1.31) | 0.29 | 1.10 (0.92, 1.32) | 0.26 |
| 2,2',4,4',5,5'-hexabromobiphenyl (PBB-153) | 482 | 1.14 (1.00, 1.29) | 0.05 | 1.12 (0.98, 1.29) | 0.10 | 1.13 (1.00, 1.27) | 0.05 |
| **Other-cause** | | | | | | | |
| Σ PBDEs | 436 | 1.06 (0.87, 1.30) | 0.53 | 1.07 (0.84, 1.36) | 0.57 | 1.05 (0.79, 1.39) | 0.74 |
| Σ PBDEs above LOD in >90% | 473 | 1.04 (0.83, 1.32) | 0.69 | 1.05 (0.81, 1.36) | 0.72 | 1.03 (0.77, 1.38) | 0.82 |
| 2,2',4,4',5,5'-hexabromodiphenyl ether (PBDE-153) | 483 | 1.09 (0.95, 1.25) | 0.19 | 1.09 (0.94, 1.27) | 0.23 | 1.08 (0.91, 1.27) | 0.37 |
| 2,2',4,4',6-pentabromodiphenyl ether (PBDE-100) | 483 | 1.02 (0.81, 1.28) | 0.88 | 1.01 (0.78, 1.31) | 0.94 | 1.01 (0.76, 1.33) | 0.97 |
| 2,2',4,4'-tetrabromodiphenyl ether (PBDE-47) | 474 | 1.04 (0.86, 1.27) | 0.66 | 1.03 (0.82, 1.28) | 0.81 | 1.02 (0.79, 1.32) | 0.88 |
| 2,2',4,4',5,5'-hexabromobiphenyl (PBB-153) | 482 | 0.86 (0.61, 1.20) | 0.34 | 0.89 (0.65, 1.22) | 0.44 | 0.85 (0.60, 1.20) | 0.33 |
| Notes: CI, confidence interval; HR, hazard ratio; LOD, limit of detection; PBDEs, polybrominated diphenyl ethers | | | | | | | |
| ^a^Weighted to consider complex survey design | | | | | |  |  |
| ^b^Adjusted for age, gender, race/ethnicity, education, and smoking status | | | | | |  |  |
| ^c^Adjusted for age, gender, race/ethnicity, BMI, poverty income ratio, education, smoking status, and alcohol consumption | | | | | | |  |

**Table S7. Associations between a one standard deviation unit increase in serum PFAS measures and all-cause, cancer, heart/cerebrovascular diseases, and other-cause mortality with varying levels of adjustment**

| **Mortality/Exposure** (ng/g) | **N** | **Age Adjusted HR (95% CI)^a^** | **p-value^a^** | **Primary Analyses HR (95% CI)^ab^** | **p-value^ab^** | **Additionally Adjusted HR (95% CI)^ac^** | **p-value^ac^** |
| --- | --- | --- | --- | --- | --- | --- | --- |
| **All-Cause** | | | | | | | |
| Perfluorooctane sulfonic acid (PFOS) | 1036 | 0.93 (0.83, 1.03) | 0.17 | 0.91 (0.80, 1.03) | 0.12 | 0.91 (0.80, 1.03) | 0.13 |
| Perfluorooctanoic acid (PFOA) | 1032 | 0.94 (0.83, 1.06) | 0.31 | 0.93 (0.82, 1.06) | 0.27 | 0.93 (0.82, 1.07) | 0.29 |
| Perfluorononanoic acid (PFNA) | 1043 | 0.95 (0.83, 1.09) | 0.49 | 0.92 (0.80, 1.07) | 0.26 | 0.92 (0.80, 1.07) | 0.26 |
| Perfluorohexane sulfonic acid (PFHxS) | 1043 | 0.88 (0.72, 1.07) | 0.19 | 0.88 (0.72, 1.08) | 0.21 | 0.89 (0.73, 1.08) | 0.22 |
| **Cancer** | | | | | | | |
| Perfluorooctane sulfonic acid (PFOS) | 1036 | 1.02 (0.90, 1.15) | 0.78 | 1.01 (0.86, 1.19) | 0.88 | 1.02 (0.86, 1.22) | 0.79 |
| Perfluorooctanoic acid (PFOA) | 1032 | 0.95 (0.81, 1.11) | 0.50 | 0.94 (0.80, 1.11) | 0.45 | 0.94 (0.78, 1.13) | 0.51 |
| Perfluorononanoic acid (PFNA) | 1043 | 0.93 (0.76, 1.13) | 0.45 | 0.89 (0.72, 1.09) | 0.25 | 0.89 (0.71, 1.12) | 0.32 |
| Perfluorohexane sulfonic acid (PFHxS) | 1043 | 1.03 (0.74, 1.44) | 0.85 | 1.06 (0.73, 1.54) | 0.74 | 1.06 (0.73, 1.54) | 0.77 |
| **Heart/cerebrovascular diseases** | | | | | | | |
| Perfluorooctane sulfonic acid (PFOS) | 1036 | 0.89 (0.70, 1.13) | 0.33 | 0.85 (0.65, 1.12) | 0.24 | 0.86 (0.65, 1.13) | 0.26 |
| Perfluorooctanoic acid (PFOA) | 1032 | 0.99 (0.83, 1.18) | 0.91 | 0.98 (0.81, 1.17) | 0.79 | 0.98 (0.82, 1.17) | 0.85 |
| Perfluorononanoic acid (PFNA) | 1043 | 1.06 (0.91, 1.24) | 0.46 | 1.03 (0.88, 1.22) | 0.69 | 1.03 (0.87, 1.22) | 0.72 |
| Perfluorohexane sulfonic acid (PFHxS) | 1043 | 0.76 (0.53, 1.10) | 0.14 | 0.76 (0.54, 1.08) | 0.12 | 0.77 (0.55, 1.08) | 0.13 |
| **Other-cause** | | | | | | | |
| Perfluorooctane sulfonic acid (PFOS) | 1036 | 0.90 (0.75, 1.08) | 0.25 | 0.89 (0.73, 1.09) | 0.26 | 0.89 (0.73, 1.09) | 0.26 |
| Perfluorooctanoic acid (PFOA) | 1032 | 0.91 (0.72, 1.15) | 0.42 | 0.90 (0.71, 1.15) | 0.39 | 0.91 (0.72, 1.14) | 0.40 |
| Perfluorononanoic acid (PFNA) | 1043 | 0.90 (0.69, 1.17) | 0.42 | 0.87 (0.66, 1.14) | 0.30 | 0.87 (0.66, 1.14) | 0.30 |
| Perfluorohexane sulfonic acid (PFHxS) | 1043 | 0.85 (0.67, 1.08) | 0.18 | 0.86 (0.68, 1.09) | 0.20 | 0.86 (0.68, 1.09) | 0.22 |
| Notes: CI, confidence interval; HR, hazard ratio; PFAS, per- and polyfluoroalkyl substance | | | | | | | |
| ^a^Weighted to consider complex survey design | |  |  |  |  |  |  |
| ^b^Adjusted for age, gender, race/ethnicity, education, and smoking status | | | |  |  |  |  |
| ^c^Adjusted for age, gender, race/ethnicity, BMI, poverty income ratio, education, smoking status, and alcohol consumption | | | | | | |  |

**Table S8. Associations between a one standard deviation unit increase in serum PCB measures and all-cause, cancer, heart/cerebrovascular diseases, and other-cause mortality with varying levels of adjustment**

| **Mortality/Exposure** (ng/g of lipid) | **N** | **Age Adjusted HR (95% CI)^a^** | **p-value^a^** | **Primary Analyses**  **HR (95% CI)^ab^** | **p-value^ab^** | **Additionally Adjusted HR (95% CI)^ac^** | **p-value^ac^** |
| --- | --- | --- | --- | --- | --- | --- | --- |
| **All-Cause** | | | | | | | |
| Σ TEF Dioxin-like PCBs above LOD in >90% | 420 | 0.92 (0.77, 1.11) | 0.36 | 0.99 (0.80, 1.23) | 0.92 | 1.01 (0.83, 1.23) | 0.90 |
| Σ TEF Dioxin-like PCBs | 385 | 0.95 (0.79, 1.15) | 0.59 | 1.01 (0.78, 1.31) | 0.92 | 1.03 (0.82, 1.30) | 0.76 |
| Σ Non-dioxin-like PCBs above LOD in >90% | 413 | 0.99 (0.81, 1.22) | 0.95 | 0.97 (0.76, 1.24) | 0.81 | 1.01 (0.79, 1.30) | 0.90 |
| Σ Dioxin-like PCBs above LOD in >90% | 414 | 0.84 (0.69, 1.03) | 0.09 | 0.91 (0.77, 1.08) | 0.27 | 0.92 (0.78, 1.09) | 0.31 |
| Σ Non-dioxin-like PCBs | 405 | 0.97 (0.78, 1.19) | 0.73 | 0.94 (0.72, 1.22) | 0.61 | 0.98 (0.76, 1.27) | 0.87 |
| Σ Dioxin-like PCBs | 380 | 0.89 (0.73, 1.08) | 0.21 | 0.94 (0.74, 1.18) | 0.55 | 0.95 (0.76, 1.18) | 0.61 |
| **Cancer** | | | | | | | |
| Σ TEF Dioxin-like PCBs above LOD in >90% | 420 | 0.87 (0.41, 1.84) | 0.69 | 0.97 (0.50, 1.87) | 0.91 | 0.99 (0.49, 2.01) | 0.98 |
| Σ TEF Dioxin-like PCBs | 385 | 0.90 (0.49, 1.63) | 0.71 | 0.95 (0.52, 1.75) | 0.87 | 0.95 (0.48, 1.85) | 0.87 |
| Σ Non-dioxin-like PCBs above LOD in >90% | 413 | 1.01 (0.62, 1.63) | 0.98 | 0.96 (0.58, 1.59) | 0.87 | 0.99 (0.65, 1.51) | 0.96 |
| Σ Dioxin-like PCBs above LOD in >90% | 414 | 0.74 (0.31, 1.77) | 0.48 | 0.85 (0.41, 1.76) | 0.63 | 0.82 (0.32, 2.10) | 0.67 |
| Σ Non-dioxin-like PCBs | 405 | 0.85 (0.43, 1.68) | 0.62 | 0.83 (0.42, 1.65) | 0.57 | 0.86 (0.46, 1.60) | 0.60 |
| Σ Dioxin-like PCBs | 380 | 0.80 (0.4, 1.61) | 0.51 | 0.87 (0.45, 1.70) | 0.67 | 0.84 (0.38, 1.85) | 0.65 |
| **Heart/cerebrovascular diseases** | | | | | | | |
| Σ TEF Dioxin-like PCBs above LOD in >90% | 420 | 0.88 (0.66, 1.18) | 0.37 | 0.96 (0.70, 1.32) | 0.78 | 0.98 (0.71, 1.33) | 0.87 |
| Σ TEF Dioxin-like PCBs | 385 | 0.92 (0.67, 1.27) | 0.59 | 0.96 (0.66, 1.38) | 0.80 | 0.98 (0.70, 1.37) | 0.89 |
| Σ Non-dioxin-like PCBs above LOD in >90% | 413 | 0.98 (0.70, 1.37) | 0.89 | 1.11 (0.72, 1.71) | 0.62 | 1.16 (0.74, 1.83) | 0.49 |
| Σ Dioxin-like PCBs above LOD in >90% | 414 | 0.86 (0.62, 1.19) | 0.35 | 0.94 (0.66, 1.35) | 0.72 | 0.96 (0.67, 1.37) | 0.79 |
| Σ Non-dioxin-like PCBs | 405 | 0.98 (0.69, 1.38) | 0.89 | 1.10 (0.71, 1.70) | 0.65 | 1.15 (0.73, 1.81) | 0.53 |
| Σ Dioxin-like PCBs | 380 | 0.88 (0.64, 1.22) | 0.43 | 0.92 (0.61, 1.40) | 0.68 | 0.94 (0.60, 1.46) | 0.77 |
| **Other-cause** | | | | | | | |
| Σ TEF Dioxin-like PCBs above LOD in >90% | 420 | 0.97 (0.73, 1.27) | 0.79 | 1.03 (0.80, 1.31) | 0.82 | 1.04 (0.82, 1.32) | 0.71 |
| Σ TEF Dioxin-like PCBs | 385 | 0.99 (0.75, 1.32) | 0.96 | 1.08 (0.83, 1.39) | 0.55 | 1.11 (0.86, 1.42) | 0.41 |
| Σ Non-dioxin-like PCBs above LOD in >90% | 413 | 1.00 (0.79, 1.27) | 0.99 | 0.91 (0.72, 1.15) | 0.41 | 0.96 (0.74, 1.24) | 0.72 |
| Σ Dioxin-like PCBs above LOD in >90% | 414 | 0.86 (0.62, 1.21) | 0.37 | 0.92 (0.70, 1.21) | 0.53 | 0.93 (0.73, 1.20) | 0.58 |
| Σ Non-dioxin-like PCBs | 405 | 0.99 (0.77, 1.27) | 0.95 | 0.90 (0.70, 1.15) | 0.35 | 0.94 (0.72, 1.23) | 0.64 |
| Σ Dioxin-like PCBs | 380 | 0.92 (0.68, 1.23) | 0.55 | 0.98 (0.72, 1.33) | 0.87 | 1.00 (0.75, 1.34) | 0.99 |
| Notes: CI, confidence interval; HR, hazard ratio; LOD, limit of detection; PCB, polychlorinated biphenyl; TEQ, toxic equivalency values | | | | | | | |
| ^a^Weighted to consider complex survey design |  |  |  |  |  |  |  |
| ^b^Adjusted for age, gender, race/ethnicity, education, and smoking status | | |  |  |  |  |  |
| ^c^Adjusted for age, gender, race/ethnicity, BMI, poverty income ratio, education, smoking status, and alcohol consumption | | | | | |  |  |

**Table S9. Association between a one standard deviation unit increase in serum OC pesticide measures and all-cause, cancer, heart/cerebrovascular diseases, and other-cause mortality with varying levels of adjustment**

| **Mortality/Exposure** (ng/g of lipid) | **N** | **Age Adjusted HR (95% CI)^a^** | **p-value^a^** | **Primary Analyses HR (95% CI)^ab^** | **p-value^ab^** | **Additionally Adjusted HR (95% CI)^ac^** | **p-value^ac^** |
| --- | --- | --- | --- | --- | --- | --- | --- |
| **All-Cause** | | | | | | | |
| Trans-nonachlor | 1404 | 1.04 (0.98, 1.10) | 0.18 | 1.05 (0.98, 1.12) | 0.17 | 1.04 (0.98, 1.12) | 0.20 |
| p,p'-DDE | 1411 | 1.05 (0.96, 1.14) | 0.27 | 1.07 (0.99, 1.16) | 0.09 | 1.07 (0.99, 1.16) | 0.08 |
| Oxychlordane | 1312 | 1.04 (0.98, 1.10) | 0.23 | 1.07 (0.996, 1.14) | 0.07 | 1.06 (0.99, 1.14) | 0.07 |
| β-hexachlorocyclohexane | 1397 | 1.08 (0.92, 1.26) | 0.32 | 1.18 (1.01, 1.38) | 0.04 | 1.18 (1.01, 1.38) | 0.04 |
| **Cancer** | | | | | | | |
| Trans-nonachlor | 1404 | 0.86 (0.74, 1.00) | 0.05 | 0.86 (0.72, 1.02) | 0.08 | 0.86 (0.72, 1.03) | 0.10 |
| p,p'-DDE | 1411 | 0.92 (0.73, 1.15) | 0.43 | 0.96 (0.75, 1.23) | 0.74 | 0.97 (0.76, 1.23) | 0.80 |
| Oxychlordane | 1312 | 0.81 (0.67, 0.98) | 0.03 | 0.83 (0.66, 1.04) | 0.10 | 0.84 (0.67, 1.05) | 0.12 |
| β-hexachlorocyclohexane | 1397 | 0.45 (0.19, 1.06) | 0.07 | 0.65 (0.27, 1.60) | 0.35 | 0.70 (0.30, 1.65) | 0.41 |
| **Heart/cerebrovascular diseases** | | | | | | | |
| Trans-nonachlor | 1404 | 0.96 (0.79, 1.17) | 0.68 | 0.99 (0.82, 1.19) | 0.92 | 0.98 (0.80, 1.20) | 0.85 |
| p,p'-DDE | 1411 | 0.98 (0.81, 1.19) | 0.86 | 1.05 (0.89, 1.24) | 0.54 | 1.05 (0.89, 1.23) | 0.58 |
| Oxychlordane | 1312 | 0.91 (0.73, 1.13) | 0.39 | 0.97 (0.78, 1.20) | 0.78 | 0.95 (0.75, 1.20) | 0.65 |
| β-hexachlorocyclohexane | 1397 | 0.99 (0.61, 1.61) | 0.96 | 1.24 (0.87, 1.76) | 0.23 | 1.18 (0.79, 1.77) | 0.41 |
| **Other-cause** | | | | | | | |
| Trans-nonachlor | 1404 | 1.10 (1.04, 1.17) | 0.001 | 1.11 (1.04, 1.19) | 0.002 | 1.11 (1.04, 1.18) | 0.002 |
| p,p'-DDE | 1411 | 1.10 (1.005, 1.20) | 0.04 | 1.12 (1.02, 1.22) | 0.02 | 1.12 (1.02, 1.23) | 0.02 |
| Oxychlordane | 1312 | 1.13 (1.06, 1.20) | 0.001 | 1.15 (1.06, 1.25) | 0.002 | 1.15 (1.06, 1.25) | 0.002 |
| β-hexachlorocyclohexane | 1397 | 1.20 (1.02, 1.41) | 0.03 | 1.26 (1.03, 1.53) | 0.03 | 1.25 (1.03, 1.52) | 0.03 |
| Notes: CI, confidence interval; HR, hazard ratio; DDE, dichlorodiphenyldichloroethylene; OC, organochlorine | | | | | | | |
| ^a^Weighted to consider complex survey design | | | | | | |  |
| ^b^Adjusted for age, gender, race/ethnicity, education, and smoking status | | | | | | |  |
| ^c^Adjusted for age, gender, race/ethnicity, BMI, poverty income ratio, education, smoking status, and alcohol consumption | | | | | | |  |

**Table S10. Sensitivity Analyses: Associations between a one standard deviation unit increase in serum PBDE measures and all-cause, cancer, heart/cerebrovascular diseases, and other-cause mortality**

| **Mortality/Exposure** | **N** | **Primary Analyses: Adjusted HR**  **(95% CI)^abc^** | **p-value^abc^** | **Unweighted Analyses: Adjusted HR (95% CI)^bc^** | **p-value^bc^** | **Adjusting for Lipids Rather Than Using Lipid-Adj. Analytes: Adjusted HR (95% CI)^ad^** | **p-value^ad^** |
| --- | --- | --- | --- | --- | --- | --- | --- |
| **All-Cause** | | | | | | | |
| Σ PBDEs | 436 | 1.07 (0.90, 1.27) | 0.42 | 1.10 (0.99, 1.22) | 0.09 | 1.07 (0.87, 1.32) | 0.49 |
| Σ PBDEs above LOD in >90% | 473 | 1.10 (0.93, 1.30) | 0.24 | 1.12 (1.01, 1.25) | 0.03 | 1.10 (0.92, 1.30) | 0.27 |
| 2,2',4,4',5,5'-hexabromodiphenyl ether (PBDE-153) | 483 | 1.07 (1.00, 1.14) | 0.06 | 1.04 (0.94, 1.16) | 0.44 | 1.06 (0.98, 1.14) | 0.13 |
| 2,2',4,4',6-pentabromodiphenyl ether (PBDE-100) | 483 | 1.05 (0.91, 1.23) | 0.47 | 1.08 (0.96, 1.21) | 0.21 | 1.05 (0.90, 1.22) | 0.53 |
| 2,2',4,4'-tetrabromodiphenyl ether (PBDE-47) | 474 | 1.10 (0.91, 1.33) | 0.29 | 1.14 (1.03, 1.26) | 0.01 | 1.11 (0.91, 1.35) | 0.30 |
| 2,2',4,4',5,5'-hexabromobiphenyl (PBB-153) | 482 | 1.04 (0.89, 1.21) | 0.63 | 1.07 (0.96, 1.18) | 0.23 | 1.02 (0.90, 1.15) | 0.79 |
| **Cancer** | | | | | | | |
| Σ PBDEs | 436 | 1.06 (0.82, 1.37) | 0.65 | 1.07 (0.85, 1.35) | 0.58 | 1.03 (0.72, 1.48) | 0.86 |
| Σ PBDEs above LOD in >90% | 473 | 1.15 (0.89, 1.49) | 0.26 | 1.16 (0.96, 1.40) | 0.13 | 1.15 (0.87, 1.50) | 0.30 |
| 2,2',4,4',5,5'-hexabromodiphenyl ether (PBDE-153) | 483 | 0.95 (0.80, 1.12) | 0.52 | 0.94 (0.67, 1.30) | 0.70 | 0.94 (0.79, 1.12) | 0.48 |
| 2,2',4,4',6-pentabromodiphenyl ether (PBDE-100) | 483 | 1.09 (0.87, 1.36) | 0.45 | 1.10 (0.88, 1.36) | 0.41 | 1.07 (0.85, 1.34) | 0.54 |
| 2,2',4,4'-tetrabromodiphenyl ether (PBDE-47) | 474 | 1.19 (0.91, 1.55) | 0.19 | 1.19 (1, 1.41) | 0.05 | 1.20 (0.88, 1.63) | 0.22 |
| 2,2',4,4',5,5'-hexabromobiphenyl (PBB-153) | 482 | 1.06 (0.87, 1.29) | 0.53 | 1.13 (0.97, 1.33) | 0.12 | 1.04 (0.87, 1.24) | 0.64 |
| **Heart/cerebrovascular diseases** | | | | | | | |
| Σ PBDEs | 436 | 1.09 (0.95, 1.25) | 0.20 | 1.12 (0.94, 1.32) | 0.21 | 1.11 (0.94, 1.32) | 0.21 |
| Σ PBDEs above LOD in >90% | 473 | 1.12 (0.97, 1.30) | 0.12 | 1.16 (0.98, 1.37) | 0.09 | 1.13 (0.96, 1.33) | 0.13 |
| 2,2',4,4',5,5'-hexabromodiphenyl ether (PBDE-153) | 483 | 1.11 (0.94, 1.31) | 0.19 | 1.11 (0.96, 1.28) | 0.16 | 1.10 (0.95, 1.28) | 0.18 |
| 2,2',4,4',6-pentabromodiphenyl ether (PBDE-100) | 483 | 1.08 (0.94, 1.24) | 0.25 | 1.13 (0.95, 1.34) | 0.18 | 1.08 (0.93, 1.25) | 0.32 |
| 2,2',4,4'-tetrabromodiphenyl ether (PBDE-47) | 474 | 1.10 (0.92, 1.31) | 0.29 | 1.14 (0.95, 1.36) | 0.15 | 1.13 (0.93, 1.37) | 0.21 |
| 2,2',4,4',5,5'-hexabromobiphenyl (PBB-153) | 482 | 1.12 (0.98, 1.29) | 0.10 | 1.14 (0.99, 1.31) | 0.08 | 1.04 (0.95, 1.15) | 0.36 |
| **Other-cause** | | | | | | | |
| Σ PBDEs | 436 | 1.07 (0.84, 1.36) | 0.57 | 1.10 (0.93, 1.30) | 0.25 | 1.05 (0.75, 1.49) | 0.75 |
| Σ PBDEs above LOD in >90% | 473 | 1.05 (0.81, 1.36) | 0.72 | 1.07 (0.88, 1.31) | 0.50 | 1.01 (0.73, 1.40) | 0.94 |
| 2,2',4,4',5,5'-hexabromodiphenyl ether (PBDE-153) | 483 | 1.09 (0.94, 1.27) | 0.23 | 1.02 (0.85, 1.23) | 0.81 | 1.08 (0.92, 1.28) | 0.33 |
| 2,2',4,4',6-pentabromodiphenyl ether (PBDE-100) | 483 | 1.01 (0.78, 1.31) | 0.94 | 1.01 (0.81, 1.27) | 0.90 | 0.98 (0.72, 1.35) | 0.92 |
| 2,2',4,4'-tetrabromodiphenyl ether (PBDE-47) | 474 | 1.03 (0.82, 1.28) | 0.81 | 1.09 (0.91, 1.32) | 0.34 | 0.98 (0.76, 1.28) | 0.89 |
| 2,2',4,4',5,5'-hexabromobiphenyl (PBB-153) | 482 | 0.89 (0.65, 1.22) | 0.44 | 0.88 (0.65, 1.18) | 0.39 | 0.84 (0.56, 1.27) | 0.38 |
| Notes: CI, confidence interval; HR, hazard ratio; LOD, limit of detection; PBDE, polybrominated diphenyl ether | | | | | | | |
| ^a^Weighted to consider complex survey design |  |  |  |  |  |  |  |
| ^b^Adjusted for age, gender, race/ethnicity, education, and smoking status | | |  |  |  |  |  |
| ^c^Lipid adjusted serum measurement (ng/g of lipid) |  |  |  |  |  |  |  |
| ^d^Adjusted for age, gender, race/ethnicity, education, smoking status, and lipids | | |  |  |  |  |  |

**Table S11. Sensitivity Analyses: Associations between a one standard deviation unit increase in serum PFAS measures and all-cause, cancer, heart/cerebrovascular diseases, and other-cause mortality**

| **Mortality/Exposure** | **N** | **Primary Analyses: Adjusted HR (95% CI)^ab^** | **p-value^ab^** | **Unweighted Analyses: Adjusted HR (95% CI)^b^** | **p-value^b^** | **N^c^** | **Outliers Included Analysis: Adjusted HR (95% CI)^abc^** | **p-value^abc^** |
| --- | --- | --- | --- | --- | --- | --- | --- | --- |
| **All-Cause** | | | | | |  |  |  |
| Perfluorooctane sulfonic acid (PFOS) | 1036 | 0.91 (0.80, 1.03) | 0.12 | 0.88 (0.77, 1.00) | 0.05 | 1043 | 0.89 (0.77, 1.01) | 0.08 |
| Perfluorooctanoic acid (PFOA) | 1032 | 0.93 (0.82, 1.06) | 0.27 | 0.92 (0.81, 1.04) | 0.19 | 1043 | 0.92 (0.8, 1.05) | 0.22 |
| Perfluorononanoic acid (PFNA) | 1043 | 0.92 (0.80, 1.07) | 0.26 | 0.94 (0.83, 1.05) | 0.26 |  |  |  |
| Perfluorohexane sulfonic acid (PFHxS) | 1043 | 0.88 (0.72, 1.08) | 0.21 | 0.86 (0.72, 1.02) | 0.08 |  |  |  |
| **Cancer** | | | | | |  |  |  |
| Perfluorooctane sulfonic acid (PFOS) | 1036 | 1.01 (0.86, 1.19) | 0.88 | 0.93 (0.70, 1.23) | 0.60 | 1043 | 0.98 (0.8, 1.21) | 0.87 |
| Perfluorooctanoic acid (PFOA) | 1032 | 0.94 (0.80, 1.11) | 0.45 | 0.97 (0.75, 1.25) | 0.82 | 1043 | 0.9 (0.74, 1.09) | 0.26 |
| Perfluorononanoic acid (PFNA) | 1043 | 0.89 (0.72, 1.09) | 0.25 | 0.93 (0.71, 1.21) | 0.57 |  |  |  |
| Perfluorohexane sulfonic acid (PFHxS) | 1043 | 1.06 (0.73, 1.54) | 0.74 | 0.96 (0.67, 1.37) | 0.82 |  |  |  |
| **Heart/cerebrovascular diseases** | | | | | |  |  |  |
| Perfluorooctane sulfonic acid (PFOS) | 1036 | 0.85 (0.65, 1.12) | 0.24 | 0.87 (0.68, 1.11) | 0.27 | 1043 | 0.85 (0.65, 1.12) | 0.25 |
| Perfluorooctanoic acid (PFOA) | 1032 | 0.98 (0.81, 1.17) | 0.79 | 0.98 (0.79, 1.22) | 0.89 | 1043 | 0.98 (0.83, 1.15) | 0.78 |
| Perfluorononanoic acid (PFNA) | 1043 | 1.03 (0.88, 1.22) | 0.69 | 1.04 (0.86, 1.25) | 0.69 |  |  |  |
| Perfluorohexane sulfonic acid (PFHxS) | 1043 | 0.76 (0.54, 1.08) | 0.12 | 0.84 (0.61, 1.17) | 0.31 |  |  |  |
| **Other-cause** | | | | | |  |  |  |
| Perfluorooctane sulfonic acid (PFOS) | 1036 | 0.89 (0.73, 1.09) | 0.26 | 0.86 (0.72, 1.04) | 0.12 | 1043 | 0.86 (0.7, 1.07) | 0.17 |
| Perfluorooctanoic acid (PFOA) | 1032 | 0.90 (0.71, 1.15) | 0.39 | 0.86 (0.71, 1.04) | 0.11 | 1043 | 0.9 (0.71, 1.14) | 0.37 |
| Perfluorononanoic acid (PFNA) | 1043 | 0.87 (0.66, 1.14) | 0.30 | 0.87 (0.72, 1.05) | 0.14 |  |  |  |
| Perfluorohexane sulfonic acid (PFHxS) | 1043 | 0.86 (0.68, 1.09) | 0.20 | 0.82 (0.64, 1.05) | 0.13 |  |  |  |
| Notes: CI, confidence interval; HR, hazard ratio; PFAS, per- and polyfluoroalkyl substance | | | | | |  |  |  |
| ^a^Weighted to consider complex survey design |  |  |  |  |  |  |  |  |
| ^b^Adjusted for age, gender, race/ethnicity, education, and smoking status | | |  |  |  |  |  |  |
| ^c^Outliers included in model; note outliers were only identified for PFOS and PFOA, but not PFNA or PFHxS | | | | | | | | |

**Table S12. Sensitivity Analyses: Associations between a one standard deviation unit increase in serum PCB measures and all-cause, cancer, heart/cerebrovascular diseases, and other-cause mortality**

| **Mortality/Exposure** | **N** | **Primary Analyses: Adjusted HR**  **(95% CI)^abc^** | **p-value^abc^** | | **Unweighted Analyses: Adjusted HR (95% CI)^bc^** | | **p-value^bc^** | | **Adjusting for Lipids Rather Than Using Lipid-Adj. Analytes: Adjusted HR (95% CI)^ad^** | | **p-value^ad^** | |  |
| --- | --- | --- | --- | --- | --- | --- | --- | --- | --- | --- | --- | --- | --- |
| **All-Cause** | | | | | | | | | | | | | |
| Σ TEQ Dioxin-like PCBs above LOD in >90% | 420 | 0.99 (0.80, 1.23) | 0.92 | | 0.96 (0.84, 1.11) | | 0.61 | | 0.98 (0.82, 1.16) | | 0.79 | |  |
| Σ TEQ Dioxin-like PCBs | 385 | 1.01 (0.78, 1.31) | 0.92 | | 0.99 (0.86, 1.15) | | 0.95 | | 1.01 (0.82, 1.23) | | 0.96 | |  |
| Σ Non-dioxin-like PCBs above LOD in >90% | 413 | 0.97 (0.76, 1.24) | 0.81 | | 0.99 (0.85, 1.16) | | 0.95 | | 0.97 (0.76, 1.24) | | 0.80 | |  |
| Σ Dioxin-like PCBs above LOD in >90% | 414 | 0.91 (0.77, 1.08) | 0.27 | | 0.89 (0.77, 1.04) | | 0.15 | | 0.88 (0.73, 1.06) | | 0.16 | |  |
| Σ Non-dioxin-like PCBs | 405 | 0.94 (0.72, 1.22) | 0.61 | | 0.96 (0.82, 1.12) | | 0.60 | | 0.94 (0.72, 1.22) | | 0.61 | |  |
| Σ Dioxin-like PCBs | 380 | 0.94 (0.74, 1.18) | 0.55 | | 0.92 (0.80, 1.07) | | 0.29 | | 0.91 (0.73, 1.12) | | 0.34 | |  |
| **Cancer** | | | | | | | | | | | | | |
| Σ TEQ Dioxin-like PCBs above LOD in >90% | 420 | 0.97 (0.50, 1.87) | 0.91 | | 0.92 (0.65, 1.30) | | 0.63 | | 1.00 (0.60, 1.66) | | 1.00 | |  |
| Σ TEQ Dioxin-like PCBs | 385 | 0.95 (0.52, 1.75) | 0.87 | | 0.94 (0.67, 1.33) | | 0.72 | | 1.00 (0.61, 1.65) | | 1.00 | |  |
| Σ Non-dioxin-like PCBs above LOD in >90% | 413 | 0.96 (0.58, 1.59) | 0.87 | | 1.00 (0.71, 1.41) | | 0.98 | | 0.98 (0.66, 1.44) | | 0.91 | |  |
| Σ Dioxin-like PCBs above LOD in >90% | 414 | 0.85 (0.41, 1.76) | 0.63 | | 0.82 (0.53, 1.25) | | 0.36 | | 0.80 (0.41, 1.54) | | 0.47 | |  |
| Σ Non-dioxin-like PCBs | 405 | 0.83 (0.42, 1.65) | 0.57 | | 0.81 (0.52, 1.26) | | 0.35 | | 0.87 (0.51, 1.46) | | 0.57 | |  |
| Σ Dioxin-like PCBs | 380 | 0.87 (0.45, 1.70) | 0.67 | | 0.86 (0.59, 1.25) | | 0.43 | | 0.83 (0.48, 1.42) | | 0.47 | |  |
| **Heart/cerebrovascular diseases** | | | | | | | | | | | | | |
| Σ TEQ Dioxin-like PCBs above LOD in >90% | 420 | 0.96 (0.70, 1.32) | 0.78 | | 0.95 (0.73, 1.24) | | 0.73 | | 0.98 (0.71, 1.34) | | 0.89 | |  |
| Σ TEQ Dioxin-like PCBs | 385 | 0.96 (0.66, 1.38) | 0.80 | | 0.98 (0.75, 1.27) | | 0.85 | | 1.00 (0.69, 1.45) | | 1.00 | |  |
| Σ Non-dioxin-like PCBs above LOD in >90% | 413 | 1.11 (0.72, 1.71) | 0.62 | | 1.13 (0.87, 1.46) | | 0.37 | | 1.05 (0.64, 1.70) | | 0.84 | |  |
| Σ Dioxin-like PCBs above LOD in >90% | 414 | 0.94 (0.66, 1.35) | 0.72 | | 0.93 (0.72, 1.21) | | 0.60 | | 0.94 (0.64, 1.38) | | 0.74 | |  |
| Σ Non-dioxin-like PCBs | 405 | 1.10 (0.71, 1.70) | 0.65 | | 1.13 (0.87, 1.45) | | 0.36 | | 1.01 (0.60, 1.72) | | 0.96 | |  |
| Σ Dioxin-like PCBs | 380 | 0.92 (0.61, 1.4) | 0.68 | | 0.93 (0.72, 1.21) | | 0.60 | | 0.92 (0.60, 1.43) | | 0.70 | |  |
| **Other-cause** | | | | | | | | | | | | | |
| Σ TEQ Dioxin-like PCBs above LOD in >90% | 420 | 1.03 (0.80, 1.31) | 0.82 | | 0.99 (0.82, 1.19) | | 0.89 | | 1.03 (0.83, 1.26) | | 0.80 | |  |
| Σ TEQ Dioxin-like PCBs | 385 | 1.08 (0.83, 1.39) | 0.55 | | 1.02 (0.84, 1.24) | | 0.82 | | 1.09 (0.83, 1.41) | | 0.52 | |  |
| Σ Non-dioxin-like PCBs above LOD in >90% | 413 | 0.91 (0.72, 1.15) | 0.41 | | 0.92 (0.74, 1.15) | | 0.47 | | 0.93 (0.73, 1.18) | | 0.53 | |  |
| Σ Dioxin-like PCBs above LOD in >90% | 414 | 0.92 (0.70, 1.21) | 0.53 | | 0.89 (0.72, 1.1) | | 0.29 | | 0.88 (0.68, 1.15) | | 0.33 | |  |
| Σ Non-dioxin-like PCBs | 405 | 0.90 (0.70, 1.15) | 0.35 | | 0.91 (0.73, 1.14) | | 0.42 | | 0.92 (0.71, 1.19) | | 0.50 | |  |
| Σ Dioxin-like PCBs | 380 | 0.98 (0.72, 1.33) | 0.87 | | 0.93 (0.76, 1.15) | | 0.53 | | 0.94 (0.71, 1.25) | | 0.64 | |  |
| Notes: CI, confidence interval; HR, hazard ratio; LOD, limit of detection; PCB, polychlorinated biphenyl; TEQ, toxic equivalency values | | | | | | | | | | | | |  |
| ^a^Weighted to consider complex survey design |  |  |  | |  | |  | |  | |  | |  |
| ^b^Adjusted for age, gender, race/ethnicity, education, and smoking status | | | |  | |  | |  | |  | |  | |
| ^c^Lipid adjusted serum measurement (ng/g of lipid) |  |  |  | |  | |  | |  | |  | |  |
| ^d^Adjusted for age, gender, race/ethnicity, education, smoking status, and lipids | | | |  | |  | |  | |  | |  | |

**Table S13. Sensitivity Analyses: Associations between a one standard deviation unit increase in serum OC Pesticide measures and all-cause, cancer, heart/cerebrovascular diseases, and other-cause mortality**

| **Mortality/Exposure** | **N** | **Primary Analyses: Adjusted HR**  **(95% CI)^abc^** | **p-value^abc^** | **Unweighted Analyses: Adjusted HR**  **(95% CI)^bc^** | **p-value^bc^** | **Adjusting for Lipids Rather Than Using Lipid-Adj. Analytes: Adjusted HR (95% CI)^ad^** | **p-value^ad^** | **N^e^** | **Outliers Included Analysis: Adjusted HR (95% CI)^abce^** | **p-value^abce^** |
| --- | --- | --- | --- | --- | --- | --- | --- | --- | --- | --- |
| **All-Cause** | | | | | | | |  |  |  |
| Trans-nonachlor | 1404 | 1.05 (0.98, 1.12) | 0.17 | 1.07 (1.02, 1.13) | 0.01 | 1.06 (0.98, 1.13) | 0.13 | 1406 | 1.05 (0.98, 1.12) | 0.16 |
| p,p'-DDE | 1411 | 1.07 (0.99, 1.16) | 0.09 | 1.02 (0.95, 1.11) | 0.54 | 1.06 (0.96, 1.16) | 0.23 | 1416 | 1.07 (0.99, 1.16) | 0.09 |
| Oxychlordane | 1312 | 1.07 (0.996, 1.14) | 0.07 | 1.08 (1.02, 1.14) | 0.01 | 1.08 (1.01, 1.16) | 0.03 | 1316 | 1.07 (1, 1.14) | 0.04 |
| β-hexachlorocyclohexane | 1397 | 1.18 (1.01, 1.38) | 0.04 | 1.18 (1.05, 1.33) | 0.004 | 1.14 (1.00, 1.31) | 0.05 | 1400 | 1.01 (0.96, 1.06) | 0.79 |
| **Cancer** | | | | | | | |  |  |  |
| Trans-nonachlor | 1404 | 0.86 (0.72, 1.02) | 0.08 | 0.93 (0.80, 1.09) | 0.37 | 0.83 (0.68, 1.00) | 0.05 | 1406 | 0.86 (0.72, 1.02) | 0.09 |
| p,p'-DDE | 1411 | 0.96 (0.75, 1.23) | 0.74 | 0.97 (0.80, 1.19) | 0.79 | 0.93 (0.71, 1.23) | 0.61 | 1416 | 0.95 (0.74, 1.23) | 0.70 |
| Oxychlordane | 1312 | 0.83 (0.66, 1.04) | 0.10 | 0.89 (0.74, 1.07) | 0.21 | 0.79 (0.61, 1.02) | 0.07 | 1316 | 0.84 (0.67, 1.05) | 0.12 |
| β-hexachlorocyclohexane | 1397 | 0.65 (0.27, 1.60) | 0.35 | 0.88 (0.55, 1.41) | 0.59 | 0.60 (0.24, 1.53) | 0.28 | 1400 | 0.65 (0.27, 1.6) | 0.34 |
| **Heart/cerebrovascular diseases** | | | | | | | |  |  |  |
| Trans-nonachlor | 1404 | 0.99 (0.82, 1.19) | 0.92 | 1.03 (0.92, 1.15) | 0.61 | 1.01 (0.82, 1.25) | 0.92 | 1406 | 0.99 (0.82, 1.2) | 0.93 |
| p,p'-DDE | 1411 | 1.05 (0.89, 1.24) | 0.54 | 0.98 (0.83, 1.17) | 0.85 | 1.07 (0.89, 1.28) | 0.47 | 1416 | 1.05 (0.9, 1.24) | 0.52 |
| Oxychlordane | 1312 | 0.97 (0.78, 1.20) | 0.78 | 1.01 (0.89, 1.16) | 0.84 | 0.97 (0.76, 1.24) | 0.80 | 1316 | 0.98 (0.8, 1.21) | 0.84 |
| β-hexachlorocyclohexane | 1397 | 1.24 (0.87, 1.76) | 0.23 | 1.08 (0.80, 1.45) | 0.62 | 1.23 (0.90, 1.68) | 0.18 | 1400 | 1.01 (0.93, 1.1) | 0.76 |
| **Other-cause** | | | | | | | |  |  |  |
| Trans-nonachlor | 1404 | 1.11 (1.04, 1.19) | 0.002 | 1.12 (1.06, 1.19) | <0.001 | 1.12 (1.05, 1.2) | 0.002 | 1406 | 1.11 (1.04, 1.19) | 0.002 |
| p,p'-DDE | 1411 | 1.12 (1.02, 1.22) | 0.02 | 1.06 (0.97, 1.17) | 0.21 | 1.09 (0.96, 1.24) | 0.16 | 1416 | 1.12 (1.02, 1.22) | 0.02 |
| Oxychlordane | 1312 | 1.15 (1.06, 1.25) | 0.002 | 1.14 (1.08, 1.22) | <0.001 | 1.17 (1.06, 1.28) | 0.002 | 1316 | 1.15 (1.06, 1.25) | 0.001 |
| β-hexachlorocyclohexane | 1397 | 1.26 (1.03, 1.53) | 0.03 | 1.25 (1.09, 1.43) | 0.001 | 1.20 (1.01, 1.44) | 0.04 | 1400 | 1.02 (0.96, 1.08) | 0.44 |
| Notes: CI, confidence interval; DDE, dichlorodiphenyldichloroethylene; HR, hazard ratio; OC, organochlorine | | | | | | | |  |  |  |
| ^a^Weighted to consider complex survey design | | |  |  |  |  |  |  |  |  |
| ^b^Adjusted for age, gender, race/ethnicity, education, and smoking status | | | | | | | | | | |
| ^c^Lipid adjusted serum measurement (ng/g of lipid) | | |  |  |  |  |  |  |  |  |
| ^d^Adjusted for age, gender, race/ethnicity, education, smoking status, and lipids | | | | | | | | | |  |
| ^e^Outliers included in model | | | | | | | | | |  |
